# Supplementary material for: A non-image-forming visual circuit mediates the innate fear of heights in male mice
Source: Nat Commun. 2024 May 3;15:3746. doi: 10.1038/s41467-024-48147-x (PMC11068790; doi:10.1038/s41467-024-48147-x)
Supplement: Supplementary file 1 — Supplementary information [file 41467_2024_48147_MOESM1_ESM.pdf]

Supplementary information

A non-image-forming visual circuit mediates the innate fear of heights in male mice

Wei Shang, Shuangyi Xie, Wenbo Feng, Zhuangzhuang Li, Jingyan Jia, Xiaoxiao Cao, Yanting Shen, Jing Li, Haibo Shi, Yiran Gu, Shi-Jun Weng, Longnian Lin, Yi-Hsuan Pan*, Xiao-Bing Yuan*

*Correspondence to: xbyuan@brain.ecnu.edu.cn, yxpan@sat.ecnu.edu.cn

Content

Supplementary Table 1..p. 02
Supplementary Table 2..p. 03
Supplementary Figure 1...p. 04
Supplementary Figure 2...p. 05
Supplementary Figure 3...p. 06
Supplementary Figure 4...p. 07
Supplementary Figure 5...p. 09
Supplementary Figure 6...p. 10
Supplementary Figure 7...p. 11
Supplementary Figure 8...p. 12
Supplementary Figure 9...p. 13
Supplementary Figure 10...p. 14
Supplementary Figure 11...p. 15
Supplementary Figure 12...p. 16
Supplementary Movie 1...p. 17
Supplementary Movie 2...p. 17
Supplementary Movie 3...p. 17
Supplementary Movie 4...p. 17
Supplementary Movie 5...p. 17
Supplementary Movie 6...p. 17
Supplementary Movie 7...p. 17
Supplementary Movie 8...p. 17
Supplementary Movie 9...p. 17
Supplementary Movie 10...p. 17

Supplementary Table 1 Coordinates and virus used for stereotaxic injection.

**Brain region**	**Coordinates (mm)**	**Volumes (μl)**
LPMR	AP: -2.06 ML: ± 0.95 DV: -2.60	0.20
SC	AP: -4.00 ML: ± 0.45 DV: -1.80	0.40
V1	AP: -3.50 ML: ± 2.30 DV: -1.40	0.40
vLGN	AP: -2.35 ML: ± 2.50 DV: -3.60	0.15
l/vlPAG	AP: -4.25 ML: ± 0.60 DV: -2.95	0.20
BLA	AP: -1.46 ML: ± 3.50 DV: -4.75	0.20
CeA	AP: -1.22 ML: ± 2.90 DV: -4.75	0.15

**Virus**	**Supplier/Lot. number**	**Titer (μg ml^{-1})**
AAV2/9-hSyn-hM4Di-EGFP	BrainVTA, PT-0153	5.15×10^{12}
AAV2/Retro-hSyn-Cre-EGFP	Braincase, BC-0160	5.00×10^{12}
AAV2/9-hSyn-DIO-hM4Di-mCherry	Braincase, BC-0153	5.06×10^{12}
AAV2/9-hSyn-DIO-hM3Dq-mCherry	Braincase, BC-0143	5.47×10^{12}
AAV2/9-hSyn-DIO-mCherry	Braincase, BC-0025	5.65×10^{12}
AAV2/9-Vglut2-hM4Di-EGFP	BrainVTA, PT-3883	5.31×10^{12}
AAV2/9-hSyn-DIO-GCaMP6s	Braincase, BC-0238	3.02×10^{12}

Supplementary Table 2 Antibodies used for immunofluorescent staining.

Antibody	Company	Catalog number	Dilution ratio
Rabbit monoclonal anti-c-fos (9F6)	Cell Signaling Technology®	2250S	1:750
Mouse monoclonal anti-c-fos (2G9C3)	Thermo Fisher	MA1-21190	1:1000
Rabbit polyclonal anti-GABA	Sigma	A2052	1:500
Rabbit polyclonal anti-Parvalbumin	Abcam	ab11427	1:500
Goat anti-rabbit IgG H+L (Alexa Fluor® 488)	Thermo Fisher	A-11034	1:1000
Goat anti-rabbit IgG H+L (Alexa Fluor® 647)	Cell Signaling Technology®	4414S	1:1000
Goat anti-mouse IgG H+L (Alexa Fluor® 488)	Thermo Fisher	A-11029	1:1000
Goat anti-rabbit IgG H+L (Alexa Fluor® 546)	Thermo Fisher	A-11035	1:1000

Supplementary Figure 1

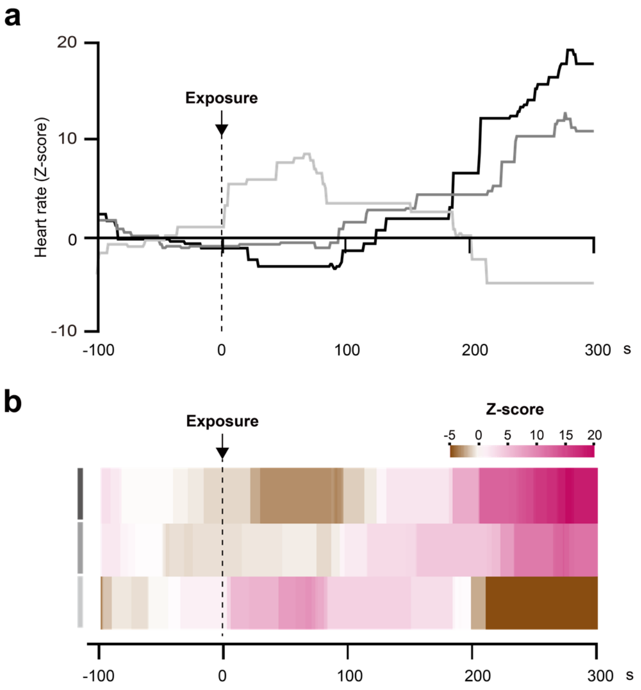

Fig. S1 The heart rate of mice increases upon height exposure. a Heart rate (HR) fluctuation over time (s, second) before and after height exposure. Z-score ((observed HR-baseline HR)/standard deviation of baseline) is used to illustrate HR changes. **b** Heatmaps of HR change over time based on the data from (**a**). Results of three mouse individuals are shown. Source data are provided as a Source Data file.

Supplementary Figure 2

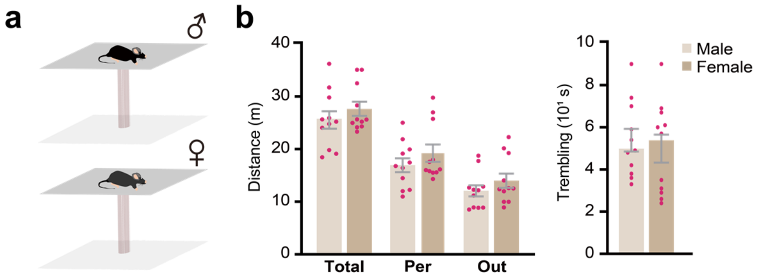

Fig. S2 Male and female mice have similar anxiety level in the OHP test. a Schematic diagram of male and female mice in the OHP test (n =11 for both male and female group). **b** No significant differences are identified between male and female mice in all parameters analyzed. Data are presented as the mean ± S.E.M. Source data are provided as a Source Data file.

Supplementary Figure 3

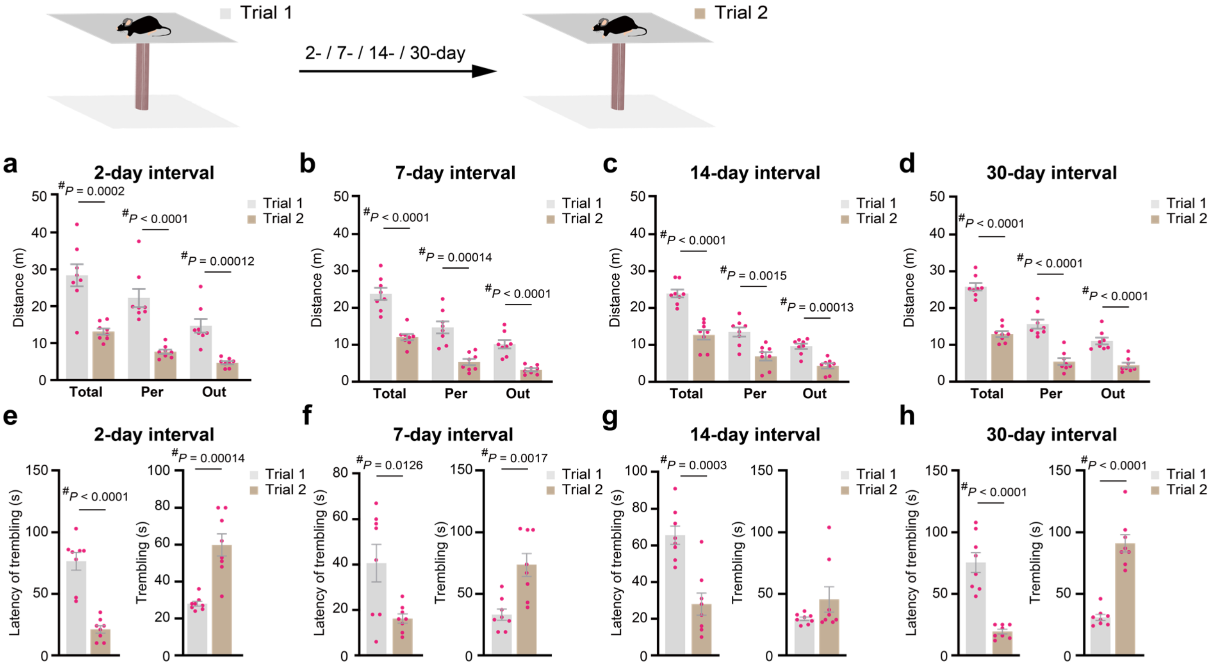

Fig. S3 Facilitation of fear of heights upon re-exposure of mice to height threat with different time intervals. a-d Comparison of the locomotion distances of mice at different regions of the OHP in two trials with different intervals (n = 8 mice/time interval, a total of 32 mice were used in this experiment). **e-h** Comparison of the latency of trembling initiation and the total duration of trembling on the OHP in two trials with different intervals. Data are presented as the mean ± S.E.M. with two-tailed Student's *t*-test ($^{\#}$). A *P* value < 0.05 is considered significant. Source data are provided as a Source Data file.

Supplementary Figure 4

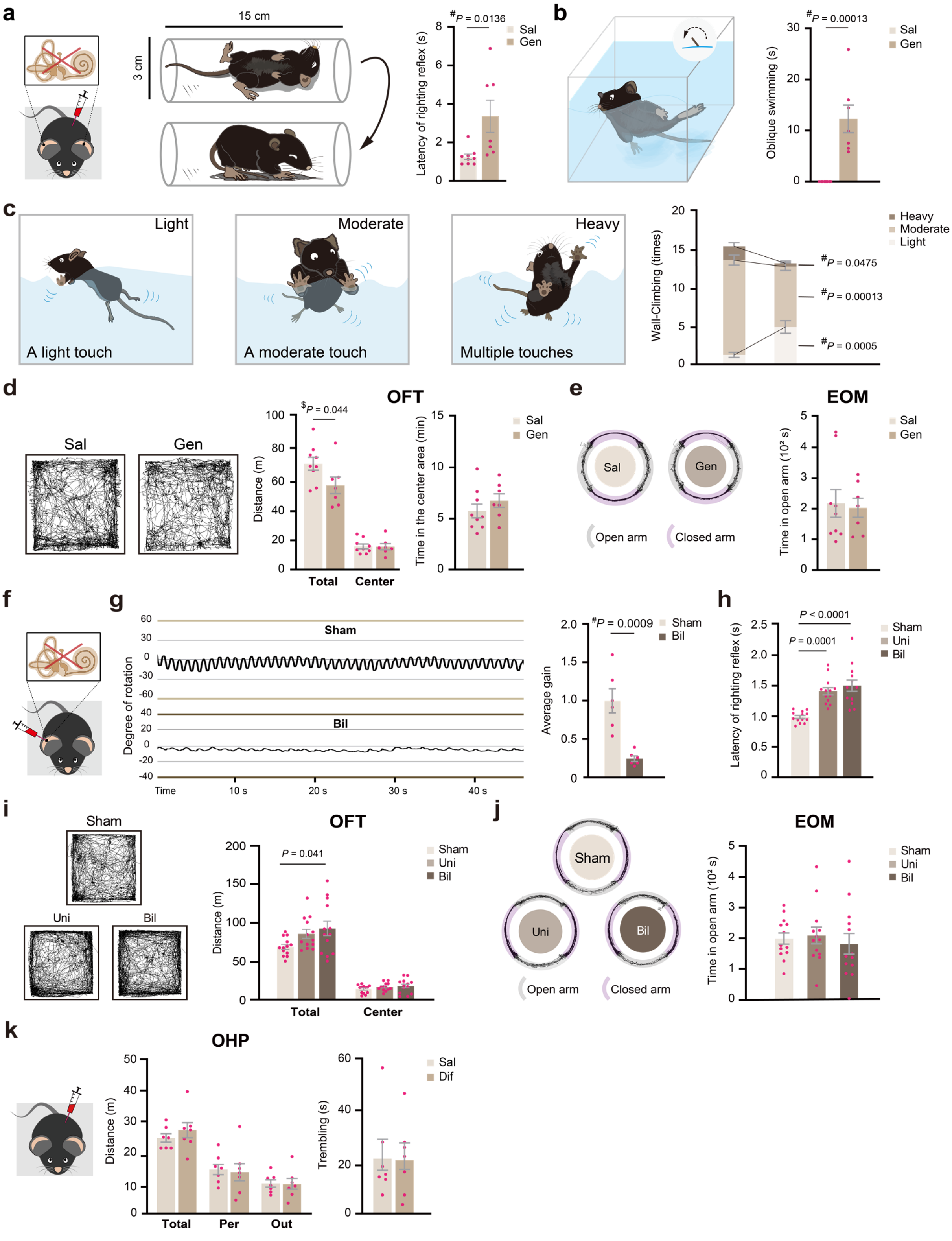

Fig. S4 Vestibular input is dispensable to fear of heights. a-c Analysis includes latency of the righting reflex (**a**), and performance in the swimming test: measured by oblique swimming time (**b**) and wall-climbing events classified as light, moderate, and heavy (**c**). **d**

Comparisons of total locomotion distance and time spent in the center during the open field test (OFT). **e** Exploration time in the open arms of the elevated O-maze test (EOM). Groups comprised 7 or 9 mice for tests (**a-e**). **f-j** Vestibulo-ocular reflex (VOR, **f**, **g**), righting reflex (**h**), OFT (**i**), and EOM (**j**) post unilateral (Uni) or bilateral (Bil) treatment. Details include a time series of eye movements and average gain values (eye movement velocity/head movement velocity) for sham and bilaterally treated groups (**f**, **g**). Group sizes were 6 mice for (**g**) and 13 mice for (**h-j**). **k** Results of OHP test for difenidol-treated mice (n = 7 mice/group). Data are presented as the mean ± S.E.M. with a One-Way ANOVA and a one-tailed ($^{\$}$) or a two-tailed ($^{\#}$) Student's *t*-test. Source data are provided as a Source Data file.

Supplementary Figure 5

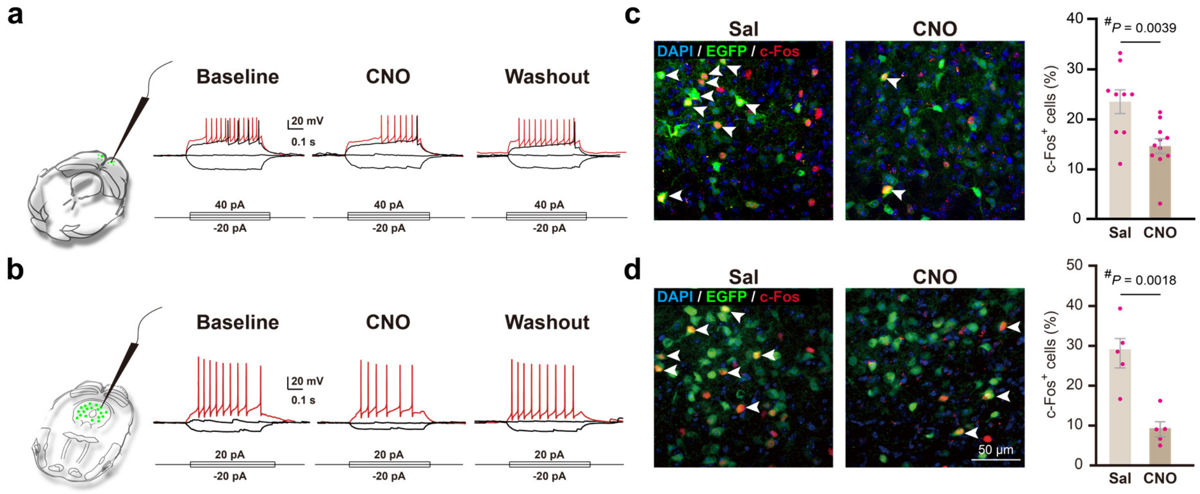

Fig. S5 Validation of the effectiveness of chemogenetic inhibition. a, b Validation of the effectiveness of chemogenetic inhibition of neuronal activities by whole-cell patch-clamp recording in brain slices from mice infected with the inhibitory virus vector hM4Di (AAV2/9-hSyn-hM4Di-EGFP) in upper layers of SC (**a**) or the l/vlPAG (**b**). Fluorescent cells that express the hM4Di vector were recorded and their firing before, during, and after washing out CNO treatment are shown. **c, d** Immunofluorescence staining of c-Fos. Coronal brain sections are from mice injected the hM4Di vector in upper layers of SC (**c**) or the l/vlPAG (**d**), treated with or without CNO (n = 2/group). The percentage of EGFP^{+} cells that are Fos^{+} with or without CNO treatment are shown. Data were analyzed using a two-tailed Student's *t*-test ($^{\#}$) and are presented as the mean ± S.E.M. Source data are provided as a Source Data file.

Supplementary Figure 6

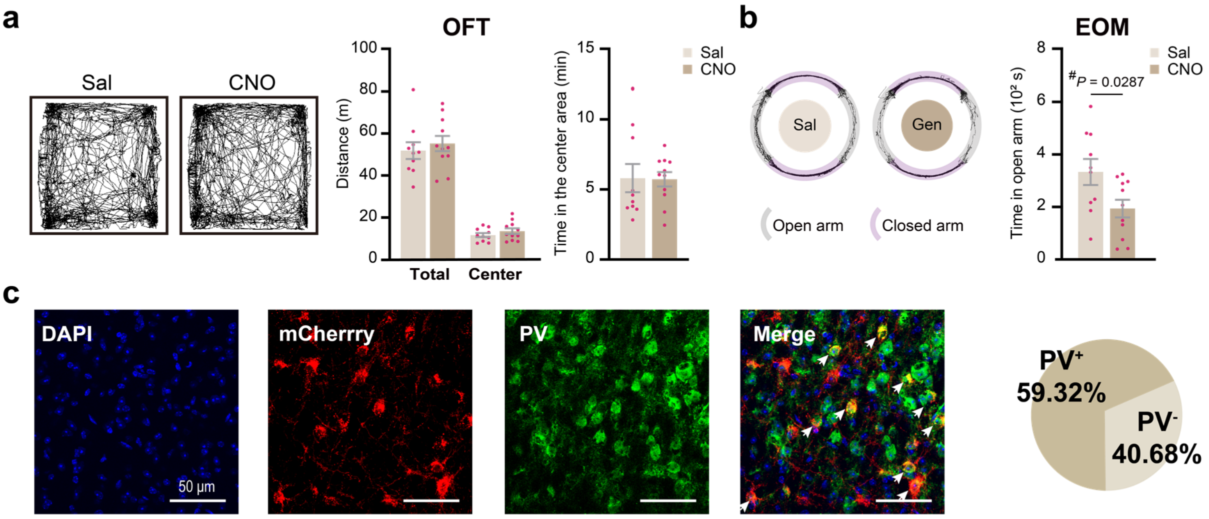

Fig. S6 Characterization of potential role of the SC. a, **b** Effects of chemogenetic inhibition of pan-neuronal activities of SC (AAV2/9-hSyn-hM4Di-EGFP) on the locomotion and anxiety level of mice in the OFT (**a**) and EOM (**b**), and groups comprised 10 mice for control and 11 mice for CNO treatment. Data are presented as the mean ± S.E.M. analyzed by a two-tailed Student's *t*-test ($^{\#}$). **c** Parvalbumin-positive (PV^{+}) neurons constitute the majority of projections from the SC to LPMR. Retrograde tracing was achieved by injecting AAV2/9-hSyn-DIO-hM4Di-mCherry (red) into the SC and AAV2/Retro-hSyn-Cre-EGFP into LPMR. SC brain slices from 2 mice were stained for PV (green, pseudo color). Source data are provided as a Source Data file.

Supplementary Figure 7

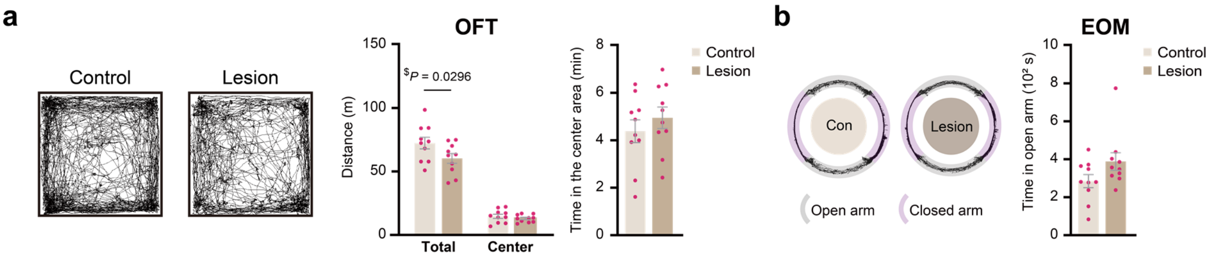

Fig. S7 V1 lesion does not affect the anxiety level of mice. a, b Effects of V1 excision on mouse behaviors in the OFT (**a**) and EOM (**b**). 10 mice/group were used. Data are presented as mean ± S.E.M. analyzed by a one-tailed ($) Student's *t*-test. Source data are provided as a Source Data file.

Supplementary Figure 8

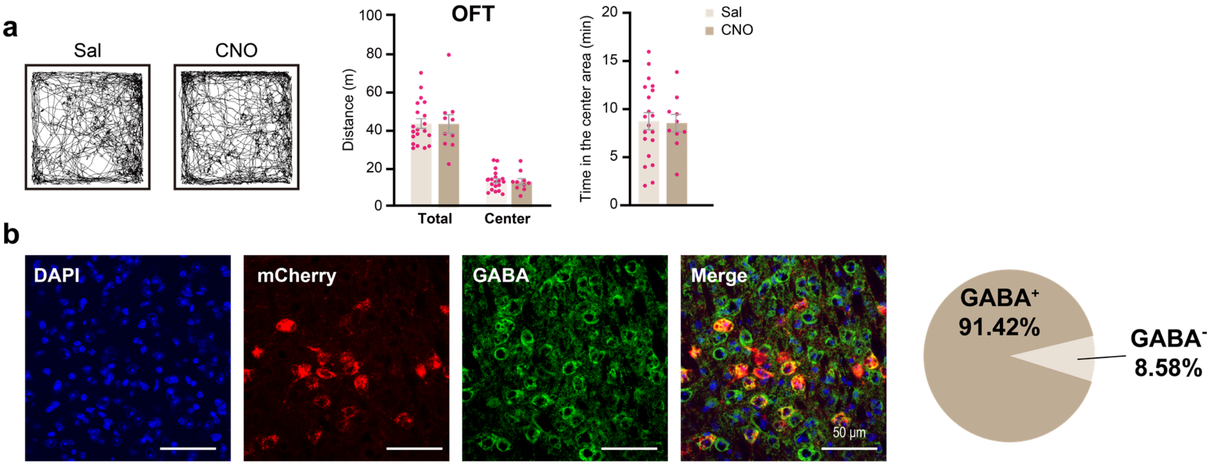

Fig. S8 Role of vLGN in the regulation of mouse behaviors. a Effect of pan-neuronal inhibition of vLGN on mouse behaviors in the OFT. **b** Immunofluorescence fate-mapping of vLGN-to-l/vlPAG projecting neurons using retrograde tracing. Retrograde tracing was achieved by injecting AAV2/Retro-hSyn-Cre-EGFP into l/vlPAG and AAV2/9-hSyn-DIO-hM4Di-mCherry (red) into vLGN. The vLGN brain slices were stained with the antibody against GABA (pseudo colored green). 20 mice for control and 10 mice for CNO treated group. The pie chart shows the proportion of $GABA^+$ cells relative to the total population of $mCherry^+$ cells (n = 3 mice). Data are presented as mean ± S.E.M. Source data are provided as a Source Data file.

Supplementary Figure 9

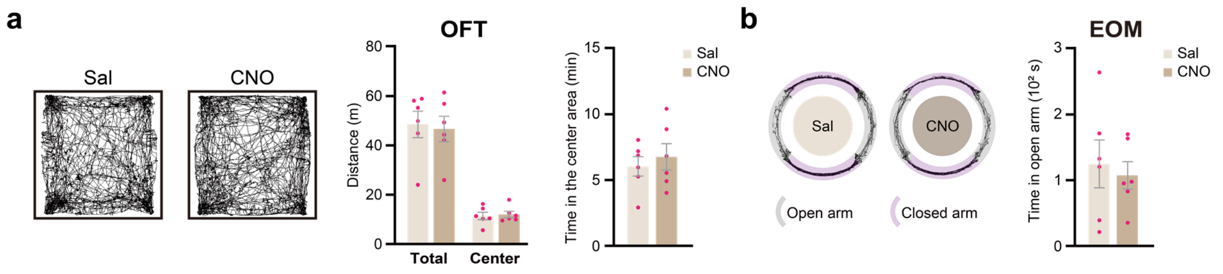

Fig. S9 Inhibition of l/vlPAG causes no significant changes in the general anxiety level. a, b Effects of chemogenetic inhibition of pan-neuronal activities of l/vlPAG on mouse behavior in the OFT (**a**) and EOM (**b**). 6 mice/group were used in this experiment. Data are presented as the mean ± S.E.M. Source data are provided as a Source Data file.

Supplementary Figure 10

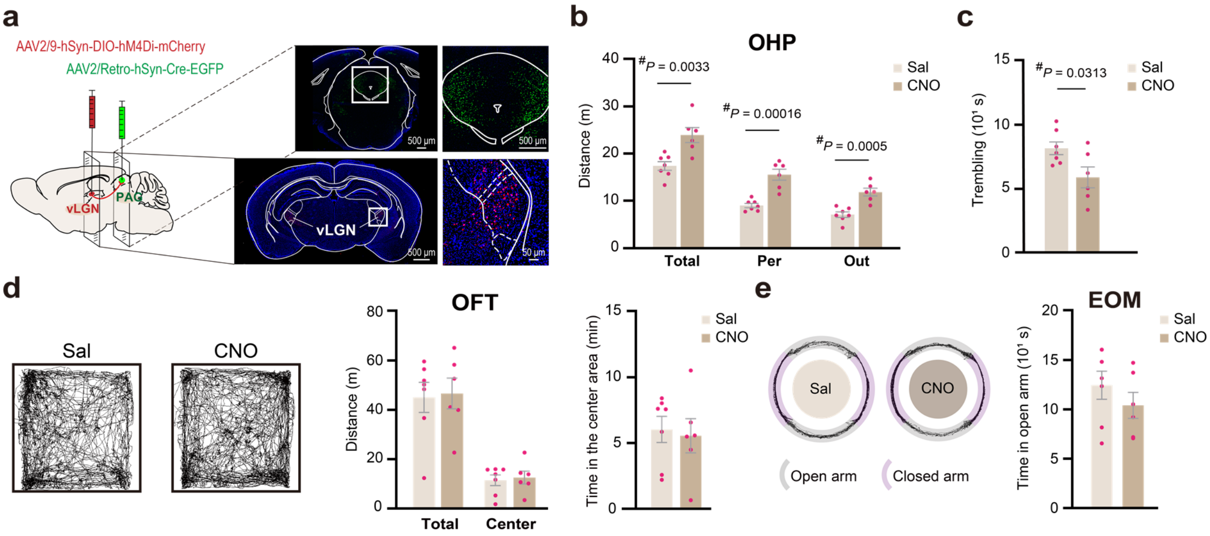

Fig. S10 Inhibition of the vLGN-PAG projecting neurons reduces fear of height. a-c Effects of chemogenetic inhibition of neurons that project from the vLGN to the l/vPAG on the fear of heights. **d, e** Effects of chemogenetic inhibition of neurons that project from vLGN to the l/vlPAG on mouse behaviors in the OFT and EOM. 7 mice for control and 6 mice for CNO treated group. Data are presented as the mean ± S.E.M. analyzed by a two-tailed Student's *t*-test (#). Source data are provided as a Source Data file.

Supplementary Figure 11

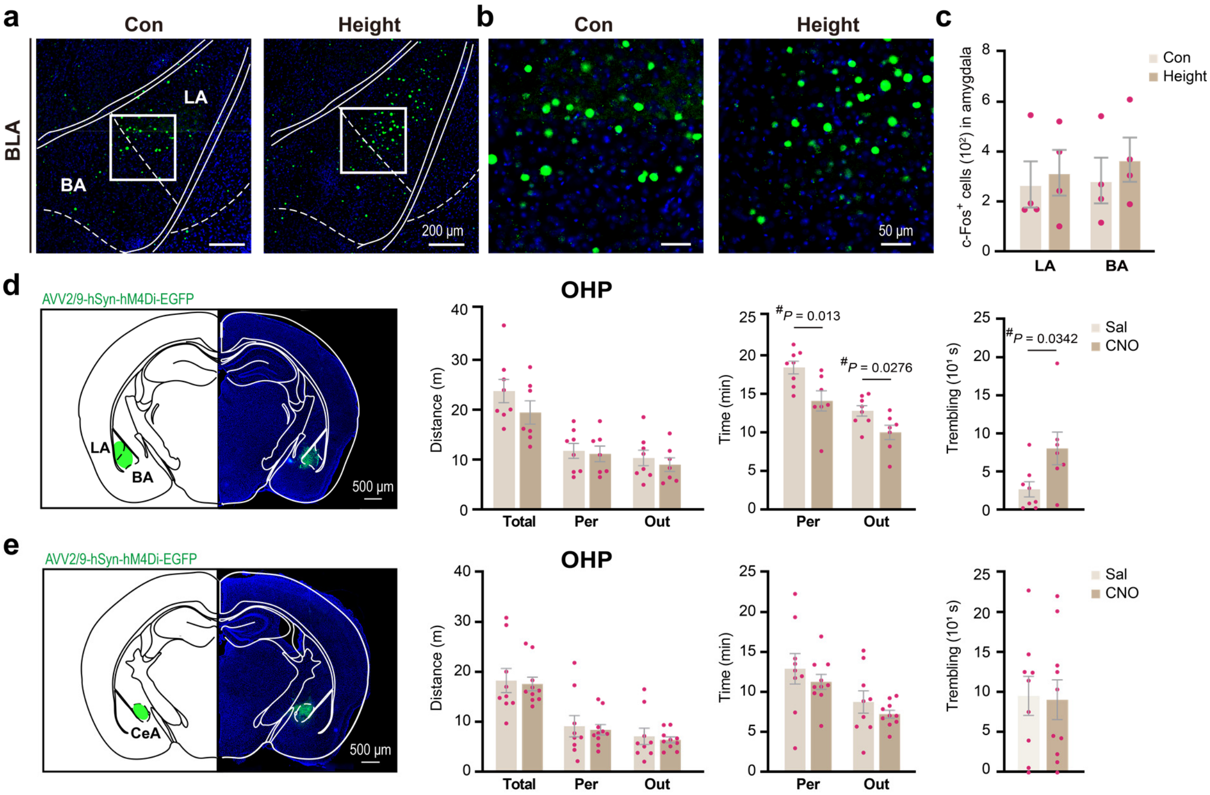

Fig. S11 The amygdala is dispensable for the fear of heights. a-c Representative images (**a**, **b**) and quantitative analysis (**c**) of c-Fos signal in different subregions of the amygdala (LA and BA) of 4 mice/group in response to height exposure. (**d**, **e**) Effects of chemogenetic inhibition of pan-neuronal activities of BLA (**d**) or CeA (**e**) on fear of heights. In (**d**), 8 mice were in the control group and 7 mice were in the CNO-treated group; in (**e**), 9 mice were in the control group and 10 mice were in the CNO-treated group. Data are presented as the mean ± S.E.M. analyzed via a two-tailed Student's *t*-test (#). Source data are provided as a Source Data file.

Supplementary Figure 12

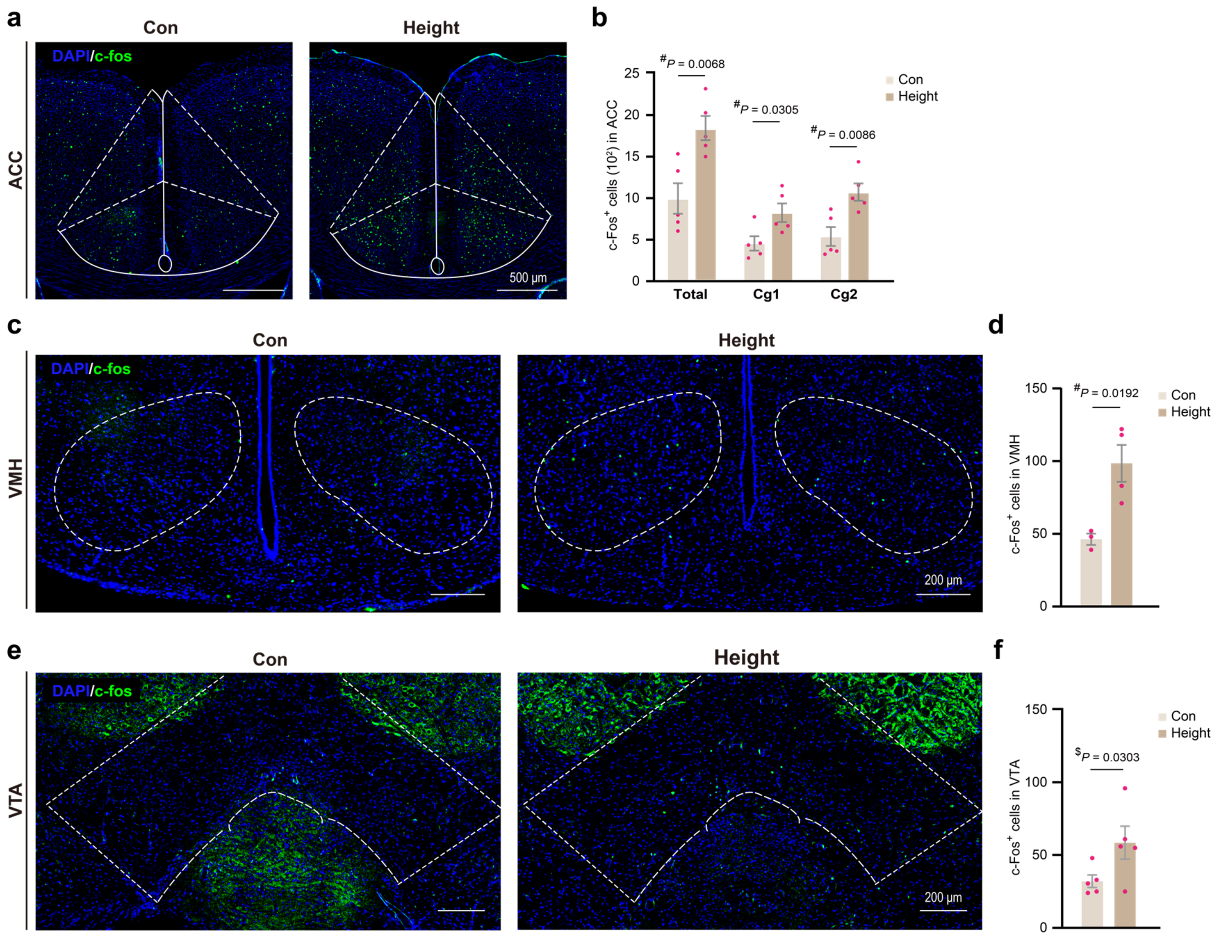

Fig. S12 Increase in the number of c-Fos⁺ cells in ACC, VMH, and VTA of mice after height exposure. Representative images (**a**, **c**, **e**) and quantitative analysis (**b**, **d**, **f**) of c-Fos⁺ cells in the anterior cingulate cortex (ACC, **a**, **b**), ventromedial hypothalamus (VMH, **c**, **d**), and ventral tegmental area (VTA, **e**, **f**) of mice in response to height exposure. In (**b**), 5 mice/group were used; in (**d**), 3 mice were in the control group and 4 mice were in the height-exposed group; in (**f**), 5 mice/group were used; Data are presented as the mean ± S.E.M. analyzed by one-tailed ($^{\$}$) or a two-tailed ($^{\#}$) Student's *t*-test. Source data are provided as a Source Data file.

Supplementary Movies

Supplementary Movie 1
Behavior of a mouse on an open high platform (OHP).

Supplementary Movie 2
Open high platform (OHP) trembling versus freezing in mice.

Supplementary Movie 3
Behavior of a mouse on an elevated platform surrounded by non-transparent walls (GWP).

Supplementary Movie 4
Behavior of a mouse on an elevated platform surrounded by transparent walls (TWP).

Supplementary Movie 5
Light (left) and dark (right) mouse behavior on an open high platform.

Supplementary Movie 6
Swimming of mice with (right) or without (left) gentamicin treatment.

Supplementary Movie 7
Wall-climbing behaviors of mice: light, moderate, and heavy.

Supplementary Movie 8
Spontaneous nystagmus and tail suspension circling in mice after 24-hour unilateral intratympanic injection of sodium arsanilate.

Supplementary Movie 9
Behavioral contrast on OHP between control (left) and l/vlPAG-Inhibited (right) mice.

Supplementary Movie 10
Chemogenetic inhibition (middle) and activation (right) of Vglut2$^+$ neurons in mouse PAG.
