# Supplementary material for: A non-image-forming visual circuit mediates the innate fear of heights in male mice
Source: Nat Commun. 2024 May 3;15:3746. doi: 10.1038/s41467-024-48147-x (PMC11068790; doi:10.1038/s41467-024-48147-x)
Supplement: Supplementary file 3 — Description of Additional Supplementary Files [file 41467_2024_48147_MOESM3_ESM.pdf]

Description of Additional Supplementary Files

Supplementary Movie 1
Behavior of a mouse on an open high platform (OHP).

Supplementary Movie 2
Open high platform (OHP) trembling versus freezing in mice.

Supplementary Movie 3
Behavior of a mouse on an elevated platform surrounded by non-transparent walls (GWP).

Supplementary Movie 4
Behavior of a mouse on an elevated platform surrounded by transparent walls (TWP).

Supplementary Movie 5
Light (left) and dark (right) mouse behavior on an open high platform.

Supplementary Movie 6
Swimming of mice with (right) or without (left) gentamicin treatment.

Supplementary Movie 7
Wall-climbing behaviors of mice: light, moderate, and heavy.

Supplementary Movie 8
Spontaneous nystagmus and tail suspension circling in mice after 24-hour unilateral intratympanic injection of sodium arsanilate.

Supplementary Movie 9
Behavioral contrast on OHP between control (left) and l/vlPAG-Inhibited (right) mice.

Supplementary Movie 10
Chemogenetic inhibition (middle) and activation (right) of $Vglut2^{+}$ neurons in mouse PAG.
