# Supplementary material for: A non-image-forming visual circuit mediates the innate fear of heights in male mice
Source: Nat Commun. 2024 May 3;15:3746. doi: 10.1038/s41467-024-48147-x (PMC11068790; doi:10.1038/s41467-024-48147-x)
Supplement: Supplementary file 14 — Reporting Summary [file 41467_2024_48147_MOESM14_ESM.pdf]

nature portfolio

Corresponding author(s): Xiao-Bing Yuan, Yi-Hsuan Pan

Last updated by author(s): Apr 5, 2024

Reporting Summary

Nature Portfolio wishes to improve the reproducibility of the work that we publish. This form provides structure for consistency and transparency in reporting. For further information on Nature Portfolio policies, see our Editorial Policies and the Editorial Policy Checklist.

Statistics

For all statistical analyses, confirm that the following items are present in the figure legend, table legend, main text, or Methods section.

n/a	Confirmed	
☐	☒	The exact sample size (*n*) for each experimental group/condition, given as a discrete number and unit of measurement
☐	☒	A statement on whether measurements were taken from distinct samples or whether the same sample was measured repeatedly
☐	☒	The statistical test(s) used AND whether they are one- or two-sided<br>*Only common tests should be described solely by name; describe more complex techniques in the Methods section.*
☒	☐	A description of all covariates tested
☒	☐	A description of any assumptions or corrections, such as tests of normality and adjustment for multiple comparisons
☐	☒	A full description of the statistical parameters including central tendency (e.g. means) or other basic estimates (e.g. regression coefficient) AND variation (e.g. standard deviation) or associated estimates of uncertainty (e.g. confidence intervals)
☐	☒	For null hypothesis testing, the test statistic (e.g. *F*, *t*, *r*) with confidence intervals, effect sizes, degrees of freedom and *P* value noted<br>*Give P values as exact values whenever suitable.*
☒	☐	For Bayesian analysis, information on the choice of priors and Markov chain Monte Carlo settings
☒	☐	For hierarchical and complex designs, identification of the appropriate level for tests and full reporting of outcomes
☒	☐	Estimates of effect sizes (e.g. Cohen's *d*, Pearson's *r*), indicating how they were calculated

Our web collection on statistics for biologists contains articles on many of the points above.

Software and code

Policy information about availability of computer code

Data collection	Data on the locomotion of animals in behavioral assays, including the open field test, elevated O-maze test, and open high platform test, were collected and analyzed using the TopScan system (CleverSys, Inc.). In the fear conditioning test, experiments on mice were conducted in a chamber (25 × 25 × 25 cm; LE116, Panlab, Spain), and the software (Packwin, Panlab, Spain) calculated the percentage of time the animals were freezing.<br>The vestibulo-ocular reflex (VOR) of mice was assessed using a VOR testing system (GAT-MVOR943, Giant Technology Co., Ltd, Shenzhen, China), and the VOR gain (the ratio of eye movement to head movement) was determined using a custom MATLAB2019 script.<br>Micro-injections of AAV vectors were performed using a 5 mL Hamilton syringe (65460-02, with replacement needles: 65461-01 or -02) connected to the TJ-4A syringe pump (Longer Precision Pump Co., Ltd.).<br>For intra-cranial administration, a micro-injection pump (ZS100, Chonry Peristaltic Pump Co., Ltd, China) with an internal injection cannula was utilized. In the fiber photometry recordings, calcium transients were captured using a fiber photometry system (R810, RWD Life Science Co., Ltd), with GCaMP6s fluorescence excited by a 470 nm LED for calcium-dependent signals, and calcium-independent signals excited by a 410 nm LED. The LEDs alternated at 20 Hz, with emissions recorded by an sCMOS camera (Photometrics Prime) at a matching frequency.<br>Heart rate data were analyzed using VitalView 6 software for the PDT 4000HR E-Mitters system (Starr Life Sciences, USA). In whole-cell patch-clamp recordings, data were acquired and analyzed using Clampfit 11.0 software (Molecular Devices).<br>Fluorescent and bright-field images of brain sections were automatically acquired with the Tissue Cytometry Analysis System (TissueGnostics GmbH, Austria) using a 20x objective.<br>Adobe Illustrator CC 1987-2017 22.0.0 (64bit) was utilized to delineate the area of each brain region of interest for counting c-Fos+ signals.
Data analysis	Statistical analysis was conducted using GraphPad Prism 10.2.2 (397) (GraphPad Software, California, USA).

For manuscripts utilizing custom algorithms or software that are central to the research but not yet described in published literature, software must be made available to editors and reviewers. We strongly encourage code deposition in a community repository (e.g. GitHub). See the Nature Portfolio guidelines for submitting code & software for further information.

Data

Policy information about availability of data

All manuscripts must include a data availability statement. This statement should provide the following information, where applicable:

- Accession codes, unique identifiers, or web links for publicly available datasets
- A description of any restrictions on data availability
- For clinical datasets or third party data, please ensure that the statement adheres to our policy

All data for Figs. 1-6 and Supplementary Figs. 1-12, Movies 1-10, and Tables 1-2 are provided in Supplementary Information files. Raw data used to generate Figs. 1-6 and Supplementary Figs. 1-12 were included in Source Data. Source data are provided with this paper. Coordinates and virus used for stereotaxic injection were provided in Supplementary Table 1. Antibodies employed in this study were provided in Supplementary Table 2.

Research involving human participants, their data, or biological material

Policy information about studies with human participants or human data. See also policy information about sex, gender (identity/presentation), and sexual orientation and race, ethnicity and racism.

Reporting on sex and gender	This item is not relevant to our study.
Reporting on race, ethnicity, or other socially relevant groupings	This item is not relevant to our study.
Population characteristics	This item is not relevant to our study.
Recruitment	This item is not relevant to our study.
Ethics oversight	This item is not relevant to our study.

Note that full information on the approval of the study protocol must also be provided in the manuscript.

Field-specific reporting

Please select the one below that is the best fit for your research. If you are not sure, read the appropriate sections before making your selection.

☒ Life sciences ☐ Behavioural & social sciences ☐ Ecological, evolutionary & environmental sciences

For a reference copy of the document with all sections, see nature.com/documents/nr-reporting-summary-flat.pdf

Life sciences study design

All studies must disclose on these points even when the disclosure is negative.

Sample size	Animals used for each of the c-fos counting experiments, chemotherapeutic manipulation, and vestibular function assessment belonged to the control group (n ≥ 3) and high-exposure group (n ≥ 3). Mice used in behavioral studies consisted of a minimum sample size of n ≥ 5 animals, as a larger sample size (n ≥ 3) was often required to obtain reliable results (see the Source Data file).<br>Animals used in fiber photometry recording or heart rate measurement comprised a group of mice (n ≥ 3).
Data exclusions	No data were excluded from the analyses. Experiments involved in chemogenetic manipulation, data from animals with the correct expression of virus vector in the target brain region were all used in the analyses
Replication	The experiments in this study were performed independently and the use and grouping of mice were detailed in the Source Data file. In our study, we did not use the same group of mice for repeated tests of height fear, except in specified instances. Typically, for groups of 10 mice, we conducted the experiments in batches of 3 to 5 mice each, depending on the length and complexity of the experiment. Subsequently, we compiled all the data from these batches for collective analysis.
Randomization	C57BL/6J mice, Vglut2-Cre mice, and Vgat-Cre mice used in the experiment were randomly allocated into control and experimental groups as needed.
Blinding	Data analyses were carried out by experimenters blind to the experimental conditions whenever possible. In our study, the authors responsible for data analysis were not aware of the mice's group assignments to prevent bias. Different researchers conducted the experiments and performed the data analysis, allowing us to maintain blinding for most of our experiments and ensure our results are as accurate as possible. However, in experiments such as those depicted in Fig. 1a-d, which describe mouse behaviors on a platform, blinding was not feasible. In these cases, the authors conducting the experiments were also tasked with detailing each movement made by the mice. Therefore, their awareness of the mice's group allocations was unavoidable.

Reporting for specific materials, systems and methods

We require information from authors about some types of materials, experimental systems and methods used in many studies. Here, indicate whether each material, system or method listed is relevant to your study. If you are not sure if a list item applies to your research, read the appropriate section before selecting a response.

Materials & experimental systems

n/a	Involved in the study
☐	☒ Antibodies
☒	☐ Eukaryotic cell lines
☒	☐ Palaeontology and archaeology
☐	☒ Animals and other organisms
☒	☐ Clinical data
☒	☐ Dual use research of concern
☒	☐ Plants

Methods

n/a	Involved in the study
☒	☐ ChIP-seq
☒	☐ Flow cytometry
☒	☐ MRI-based neuroimaging

Antibodies

Antibodies used	Primary and secondary antibodies used for immunohistochemistry are listed in Supplementary Table 2. These include c-Fos (9F6) rabbit monoclonal antibody (Cell Signaling Technology®, 2250S), used at a dilution of 1:750; c-Fos (2G9C3) mouse monoclonal antibody (Thermo Fisher, MA1-21190), used at 1:1000; anti-Parvalbumin rabbit polyclonal antibody (Abcam, ab11427), at 1:500; and GABA rabbit polyclonal antibody (Sigma, A2052), at 1:500. The secondary antibodies used were goat anti-rabbit IgG H+L (Alexa Fluor® 488, Thermo Fisher, A-11034), goat anti-rabbit IgG H+L (Alexa Fluor® 647, Cell Signaling Technology®, 4414S), goat anti-mouse IgG H +L (Alexa Fluor® 488, Thermo Fisher, A-11029), and goat anti-rabbit IgG H+L (Alexa Fluor® 546, Thermo Fisher, A-11035), all at 1: 1000.
Validation	Antibodies employed in this study were provided in Supplementary Table 2.

Animals and other research organisms

Policy information about studies involving animals; ARRIVE guidelines recommended for reporting animal research, and Sex and Gender in Research

Laboratory animals	Two-month-old C57BL/6J mice were purchased from Shanghai Jihui Laboratory Animal Care Co., Ltd. Vglut2-Cre mice (#: 028863) and Vgat-Cre mice (#: 028862), also two months old, were obtained from the Jackson Laboratory. All mice were bred within an SPF (Specific Pathogen-Free) barrier system and were housed in groups of five per cage with free access to food and water, and subjected to a 12-hour light/dark cycle. Except for the experiments in Supplementary Fig. 2, male mice were used. These mice underwent an acclimation period of at least one week before being used in any experiments (see Methods).
Wild animals	The study did not involve wild animals.
Reporting on sex	We conducted almost all of the experiments using male mice, as we found no significant differences in responses to height between male and female mice (Fig. S2).
Field-collected samples	The study did not involve sample collected from the field.
Ethics oversight	Mouse care and experiments were performed according to the guidelines for the Care and Use of Laboratory Animals of the National Institutes of Health. All animal procedures were approved by the Animal Care and Use Committee of East China Normal University (m20210411).

Note that full information on the approval of the study protocol must also be provided in the manuscript.

Plants

Seed stocks	This item is not relevant to our study.
Novel plant genotypes	This item is not relevant to our study.
Authentication	This item is not relevant to our study.
